# Clinicopathological features and prognosis of primary membranous nephropathy in combination with crescent

**DOI:** 10.1007/s11255-022-03457-1

**Published:** 2023-01-09

**Authors:** Yan Pan, Lei Liu, Weidong Chen, Huijuan Yang, Jiqiang Zhang, Ying Wang

**Affiliations:** Nephrology Department, The First Affiliated Hospital of Bengbu Medical College, Bengbu, 233000 China

**Keywords:** Primary membranous nephropathy, Crescentic body, Clinicopathology, Prognosis, Immunosuppressive therapy

## Abstract

**Objective:**

The incidence of primary membranous nephropathy with crescentic bodies is low, but the specificity of its clinical presentation, pathology and prognosis is of great interest. In this study, we retrospectively analyzed the clinicopathological and prognostic characteristics of patients with crescentic MN in our hospital over the past 4 years.

**Methods:**

Ten patients with combined crescentic primary membranous nephropathy diagnosed by renal biopsy at our hospital from 2018 to 2021 were retrospectively analysed and compared with 39 patients with PMN (simple random sample) during the same period for clinicopathological and prognostic comparisons.

**Results:**

The 10 patients had higher 24 h urine protein quantification, creatinine levels on renal biopsy, interstitial fibrosis and tubular atrophy, and interstitial inflammatory cell infiltration than the control group (*P* < 0.05); there were no significant differences in anti-PLA2R antibodies and PLA2R staining of renal tissue (*P* > 0.05); At follow-up, the poor outcome of crescentic MN treatment and the low clinical remission rate were found, with the percentage of crescentic bodies being a factor in patient prognosis (*P* < 0.05).

**Conclusion:**

Crescentic MN has a low prevalence and maybe a specific type of PMN; it has more severe clinical symptoms and pathology than PMN, and the crescentic proportion is strongly associated with renal prognosis. Intensive treatment is recommended for these patients.

## Background

Primary membranous nephropathy (Primary membranous nephropathy, PMN) is one of the most common causes of adult nephrotic syndrome, the incidence of which has increased dramatically in China [1]. The pathology of PMN shows diffuse thickening of the glomerular capillary wall on light microscopy; immunohistochemical staining shows granular positivity for immunoglobulin and complement components; and electron microscopy shows electron-dense deposits in the subepithelium, the outer part of the glomerular basement membrane (GBM), with extensive loss of podocyte peduncles. A review of previous studies has revealed a rare pathological type of IMN—membranous nephropathy combined with crescent formation—which has somewhat expanded our knowledge of IMN further [2, 3]. However, the mechanism of crescentogenesis, its relationship with the clinical expression and pathological features and the prognosis of membranous nephropathy, has been poorly studied. To fill these gaps, members of the research group plan to analyse the clinical, pathological and prognostic aspects of patients with IMN combined with crescent formation. To address these shortcomings, members of the research team plan to analyse the clinical, pathological and prognostic aspects of patients with PMN combined with crescent formation. This study is a multi-faceted controlled study between patients with crescentic membranous nephropathy and primary membranous nephropathy at our institution during the same period of time. Through the results of the study, we hope to understand the clinical, pathological and prognostic peculiarities of patients with the rare pathological type of PMN—crescentic membranous nephropathy, to develop the most appropriate treatment and to further improve the content of knowledge in the field of PMN.

## Research methodology

### Patients

There were 330 patients with PMN confirmed by renal pathology biopsy in our nephrology department from 2018 to 2021, including 10 patients with primary membranous nephropathy with crescentic bodies, as well as 39 patients with primary membranous nephropathy were randomly selected to exclude patients with secondary MN such as hepatitis B/C virus infection, lupus, malignancy, rheumatoid arthritis, drug and heavy metal toxicity; patients with ANCA, anti-GBM antibodies, lupus or other conditions that could lead to crescentic MN were also excluded. Clinical information was collected from patients at the initial visit and during follow-up. The study was in accordance with the Declaration of Helsinki, the study was approved by the Ethics Committee of the First Affiliated Hospital of Bengbu Medical College (Lunke Approval [2020] No. 117), and all study subjects signed an informed consent form. Informed consent was obtained for tissue and blood sampling.

### Blood and body fluid tests

General information Baseline clinical data and laboratory tests were collected from PMN patients, including age, sex, weight, albumin, urinary protein (UTP), blood creatinine, and estimated glomerular filtration rate (eGFR)., uric acid, lipids. eGFR formula = 175 × Scr-1.234 × age − 0.197 [female × 0.79]) (2009 CKD-EPI formula) [4].

### Anti-PLA2R antibody test

The level of PLA2R antibody is measured by ELISA double antibody sandwich assay, following the instructions of the kit. Serum PLA2R antibody kit < 14 RU/ml is considered negative [5].

### Renal pathology

Kidney biopsy specimens were examined according to the Centre's standard operating procedures. Renal light microscopy, including hematoxylin–eosin (H&E), Jones-methylamine silver, Masson trichrome and Schiff's reagent periodate staining was performed. Pathological staging was based on Ehrenreich and Churg's criteria for stages I, II, III and IV [6], looking at the glomerulus, tubules, interstitium and small renal arteries. A semi-quantitative method was used for interstitial fibrosis. There are 4 grades according to the extent of the lesion, defined as follows: grade 1: 0 < lesion area < 5%; grade 2: lesion area 5–24%; grade 3: lesion area 25–49%; grade 4: lesion area ≥ 50% [7]. Immunofluorescence: Fresh kidney histology-1 woven specimens were embedded in OCT (optimal cutting temperature compound), made into frozen sections and cut at a thickness of 3 um on a cryostat. IgG (IgG1; IgG2; IgG3; IgG4); IgA; IgM; C3; C1q; PLA2R [8] were detected by direct immunofluorescence and the fluorescence intensity shown under the microscope could be graded from 0 to 4 + .

### Follow-up data

Serum albumin, 24 h urine protein volume, blood creatinine and eGFR were recorded during the follow-up period.

### Treatment

The use of steroids and immunosuppressants in our section was in accordance with the 2012 KDIGO. (1) Complete remission: 24-h urine protein < 0.3 g, serum albumin > 35 g/L and normal serum creatinine; (2) Partial remission: 24-h urine protein quantification < 3.5 g and >50% decrease, improved serum albumin and stable creatinine; (3) No remission means that the above criteria are not met.

### Statistical methods

Statistical analysis was performed using the statistical software SPSS 23.0. Simple random sampling was applied to randomly select patients with PMN without crescent. Normally distributed data were expressed as mean ± standard deviation (*x * ±  *s*) and *t* test was used for comparison between samples of 2 groups. Expressed as median (P25, P75), comparisons between groups were made using the Man-Whitney test, and differences in qualitative data were compared using the × 2 test. Kaplan–Meier curves were used to analyse renal regression. One-way survival analysis was performed using the log-rank test. Results were expressed as the dominance ratio (OR) or relative risk ratio (RR) and 95% confidence interval (CI). Considering the small sample size of crescentic patients, the significance level was set at *P* < 0.05 to improve the accuracy of risk prediction.

## Results

### Flow chart of patient inclusion and follow-up

**Fig. 1:**
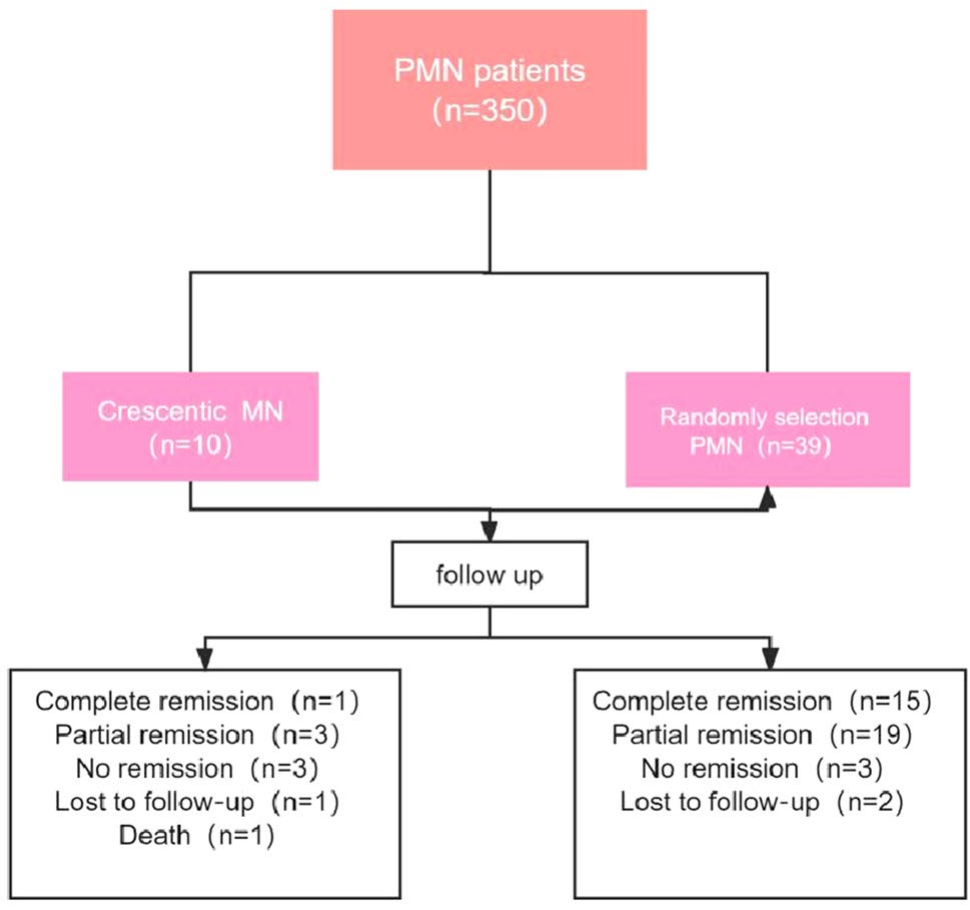
Flow chart of patient inclusion and follow up

Pathology on renal biopsy between January 2018 and June 2021 in our nephrology department showed a total of 350 cases of PMN, of which 10 were combined with crescent formation (2.86%), a low prevalence in PMN; of the 340 patients with Primary MN without crescent formation, 39 were randomly selected for relevant comparison (see Fig. 1).

### Baseline clinical features

Of the patients with neonatal MN, 8 (80%) were male, with a mean age of 50.0 years, of whom 9 (90%) had nephrotic syndrome; the patients' 24 h urine protein quantification and serum creatinine were significantly elevated, and eGFR decreased significantly, with statistically significant differences compared to the PMN group (*p* < 0.05); the differences in serum albumin and anti-PLA2R antibody levels were not statistically significant (*P* > 0.05). See Table 1 for details.

**Table 1:** Comparative analysis of the clinical characteristics of the two groups of patients

	Group		Z/χ^2^ values	*P*
	PMN (*n* = 39)	Crescentic MN (*n* = 10)		
Gender M, %	26 (66.67)	8 (80.00)	0.666	0.414
Age (years)	48 (44.0, 58.0)	50 (34.8, 60.0)	− 0.161	0.872
Body weight (kg)	66.00 (60.0, 78.0)	76.00 (71.0, 85.0)	− 2.092	0.036*
Urinary protein, g/24 h	4.17 (2.5, 5.8)	5.96 (4.0, 7.8)	− 1.997	0.046*
Albumin, g/l	24.00 (22.2, 29.0)	21.25 (19.6, 28.2)	− 1.762	0.078
Nephrotic syndrome,n (%)	23 (60.53)	9 (90.00)	3.095	0.079
Serum creatinine,umol/l	64.00 (57.50, 73.50)	88.00 (70.50, 97.00)	− 3.326	0.0009**
eGFR, mL/(min-1.73 m^2^)	113.80 (94.61, 127.21)	96.31 (72.99, 103.54)	− 2.368	0.0179*
Anti-PLA2R antibody, RU/ml	76.76 (43.3, 112.2)	100.00 (57.9, 204.8)	− 0.857	0.391
Anti-PLA2R antibody positive,* n* (%)	31 (79.49)	10 (100.00)	2.452	0.117

*P < 0.05

**P < 0.01

### Baseline pathological features in both groups

First, we found a mean percentage of crescentic glomeruli of 5.72% (range 4.89–11.07%) in patients with crescentic MN under light microscopy, with four biopsy specimens showing cellular crescents and six showing fibroblastic crescents without fibrous crescent formation, a statistically significant difference compared to the PMN group (*P* < 0.01). The group with crescents had a higher proportion of thylakoid and stromal hyperplasia, spherical and segmental sclerosis, but the difference was not statistically significant compared to the PMN group (*P* > 0.05); the interstitial inflammatory cell infiltration was heavier and the rate of tubular atrophy and interstitial fibrosis was higher (*P* < 0.01); the histopathological stage was later than that of the control group (*P* < 0.01). Second, under immunofluorescence, we compared the deposition of multiple immunoglobulins and complement in the renal tissues of the two groups. 10 patients (100%) in the crescent group had PLA2R staining deposits; at C3 and C1q staining, there was variability in deposition intensity (*P* < 0.05). See Table 2 for details.

**Table 2:** Comparative analysis of the pathological characteristics of the two groups of patients

	Group		Z/χ^2^/*t* values	*P*
	PMN (*n* = 39)	Crescentic MN (*n* = 10)		
Total no. of glomeruli	18.77 ± 7.57	18.30 ± 8.93	0.169	0.867
Mesangial cell proliferation, *n* (%)	19 (48.72)	8 (80.00)	3.15	0.076
Crescent, *n* (%)	0	5. 72%	− 6.8695	*P* < 0.001**
Cellular crescent, *n* (%)	0	0%	− 4.0746	*P* < 0.001**
Fibrocellular crescents, *n* (%)	0	4. 77%	− 5.0968	*P* < 0.001**
Glomerular sclerosis, *n* (%)	4. 35%	5. 00%	− 0.1680	0.5001
Segmental glomerular sclerosis, *n* (%)	0 (0,0)	0%	− 0.8807	0.3585
Interstitial inflammatory cell infiltration None, *n* (%)	1 (2.56)	0 (0.00)	0.262	0.609
Interstitial inflammatory cell infiltration Scattered, *n* (%)	20 (51.28)	2 (20.00)	3.148	0.076
Interstitial inflammatory cell infiltration Focal, *n* (%)	14 (35.90)	4 (40.00)	0.058	0.810
Interstitial inflammatory cell infiltration Large patch, *n* (%)	3 (7.69)	4 (40.00)	6.785	0.009**
Small arterial lesions, *n* (%)	36 (92.31)	10 (100.00)	0.819	0.365
Interstitial fibrosis and tubular atrophy,* n*%	20 (51.28)	10 (100.00)	7.957	0.005**
Grade 1: lesion area < 5%, *n* (%)	10 (25.64)	3 (30.00)	0.078	0.781
Grade 2: lesion area 5–24%, *n* (%)	21 (53.85)	5 (50.00)	0.047	0.828
Grade 3: lesion area 25–49%, *n* (%)	0 (0.00)	2 (20.00)	8.132	0.004**
MN stages I, *n* (%)	1 (2.63)	0 (0.00)	0.269	0.604
MN stages II, *n* (%)	32 (88.89)	5 (50.00)	7.521	0.006**
MN stages III, *n* (%)	4 (10.26)	5 (50.00)	8.385	0.004**
Immunofluorescence				
PLA2R	2 (± − 3)	2 (1–3)	− 0.209	0.834
IgA	0 (0–2)	0 (± − 2.0)	− 0.148	0.882
IgM	1 (0–1)	1 (0–1)	− 1.094	0.274
C3	2 (0–3)	3 (2–3)	− 3.725	*P* < 0.001**
C1q	0 (0- ±)	0(0–2)	− 1.969	0.049*
IgG	3(2–3)	3(3.0)	− 0.513	0.608
IgG1	1 (0–3)	1 (0–3)	− 0.168	0.867
IgG2	1 (0–2)	1 (1–2)	− 1.551	0.121
IgG3	0 (0–2)	1 (0–2)	− 0.743	0.457
IgG4	3 (0–4)	3 (1–4)	− 1.700	0.089

*P < 0.05

**P < 0.01

### Comparative analysis of follow-up results

37 of the 39 patients were followed up with treatment regimens that included optimised therapy (ACEI/ARB analogues), glucocorticoids combined with cyclophosphamide or neurocalcineurin inhibitors. Two patients in the PMN group were lost to follow-up and one patient in the PMN with crescent group died of a concomitant lung infection (14 months follow-up). Patients in the PMN group with crescent bodies had high creatinine and low eGFR at diagnosis, a statistically significant difference compared to the PMN group (*P* < 0.05); creatinine and eGFR at the last follow-up were not statistically significant compared to the control group (*P* > 0.05). In the PMN group, serum albumin was low and 24 h urine protein quantification was high at the last follow-up, with a statistically significant difference compared to the PMN group (*P* < 0.01); the difference between the two groups was not statistically significant at the time of diagnosis (*P* > 0.05) See Table 3.

**Table 3:** Comparative analysis of follow-up results between the two groups

	Group		*t*/*U*/χ^2^ values	*P*
	PMN (*n* = 37)	Crescentic MN (*n* = 9)		
Follow-up time, months	12.16 ± 2.11	9.33 ± 3.74	2.185	0.056
Serum creatinine, umol/L	67.8649 ± 24.8430	90.4444 ± 26.7447	− 2.4108	0.0202*
Serum creatinine at last follow-up,umol/L	71.9459 ± 42.7785	98.1111 ± 54.9214)	− 1.5565	0.1268
eGFR,mL/(min-1.73 m^2^)	111.8822 ± 28.0284	87.0244 ± 26.2725	2.4130	0.0201*
Final eGFR,mL/(min-1.73 m^2^)]	105.0022 ± 27.6555	86.4033 ± 30.9180	1.7697	0.0837
Serum albumin,g/L	24.00 (22.0,29.6)	22.20 (19.8,28.8)	115.000	0.154
Serum albumin at last follow-up,g/L	41.70 (38.9, 44.1)	27.20 (26.1, 38.7)	59.000	0.003**
Urine protein, g/24 h	4.30 (2.9, 6.2)	5.31 (3.7,7.2)	116.500	0.166
Urine protein at last follow-up, g/24 h	0.54 (0.2, 1.0)	4.02 (1.4, 5.9)	68.500	0.007**
Complete remission, *n* (%)	15 (40.54)	1 (11.11)	2.764	0.096
Partial remission, *n* (%)	19 (51.35)	3 (33.33)	0.942	0.332
No remission, *n* (%)	3 (8.11)	5 (55.56)	11.344	0.001**

*P < 0.05; **P < 0.01

**Fig. 2:**
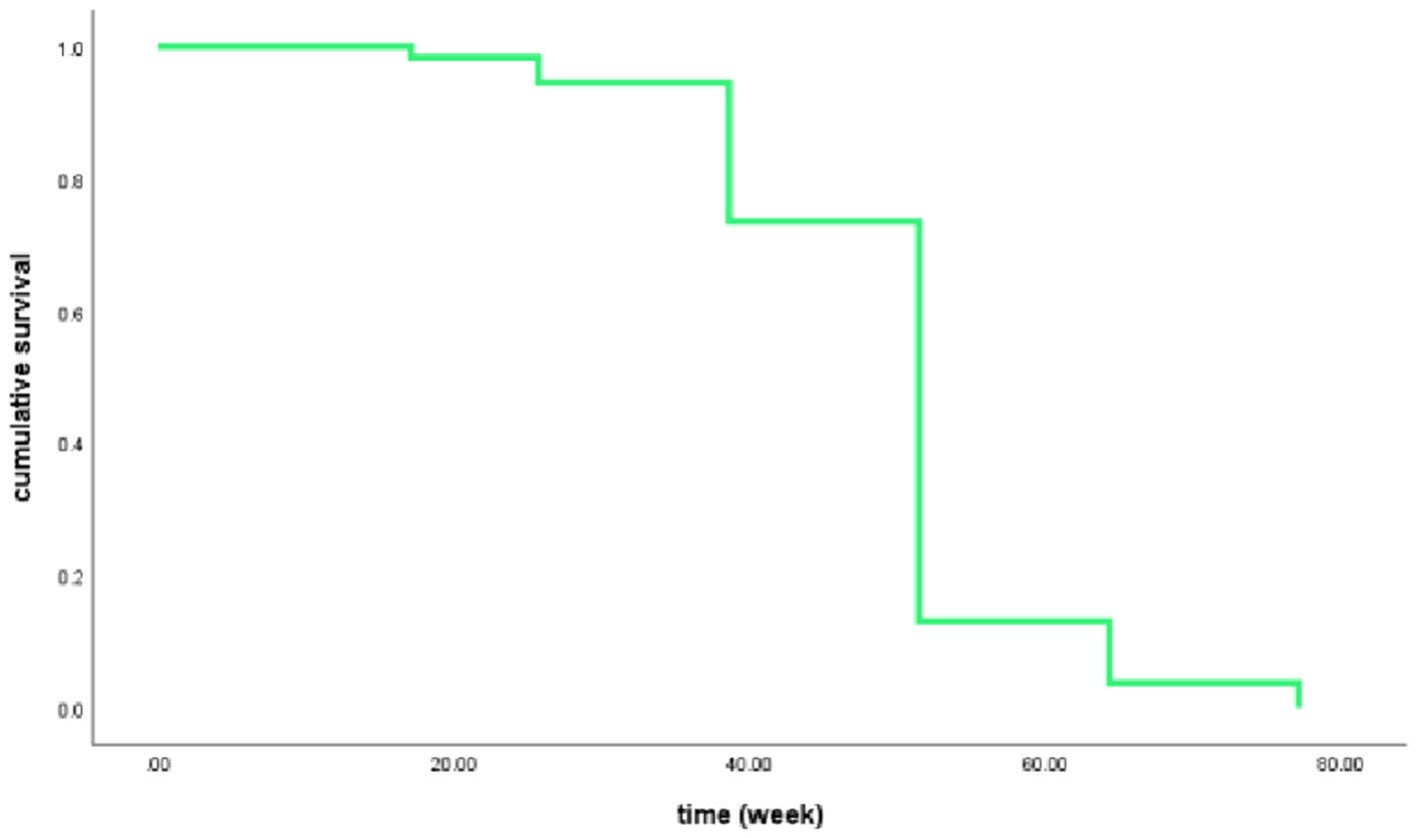
Plot of the survival analysis function from the mean of the crescent

**Fig. 3:**
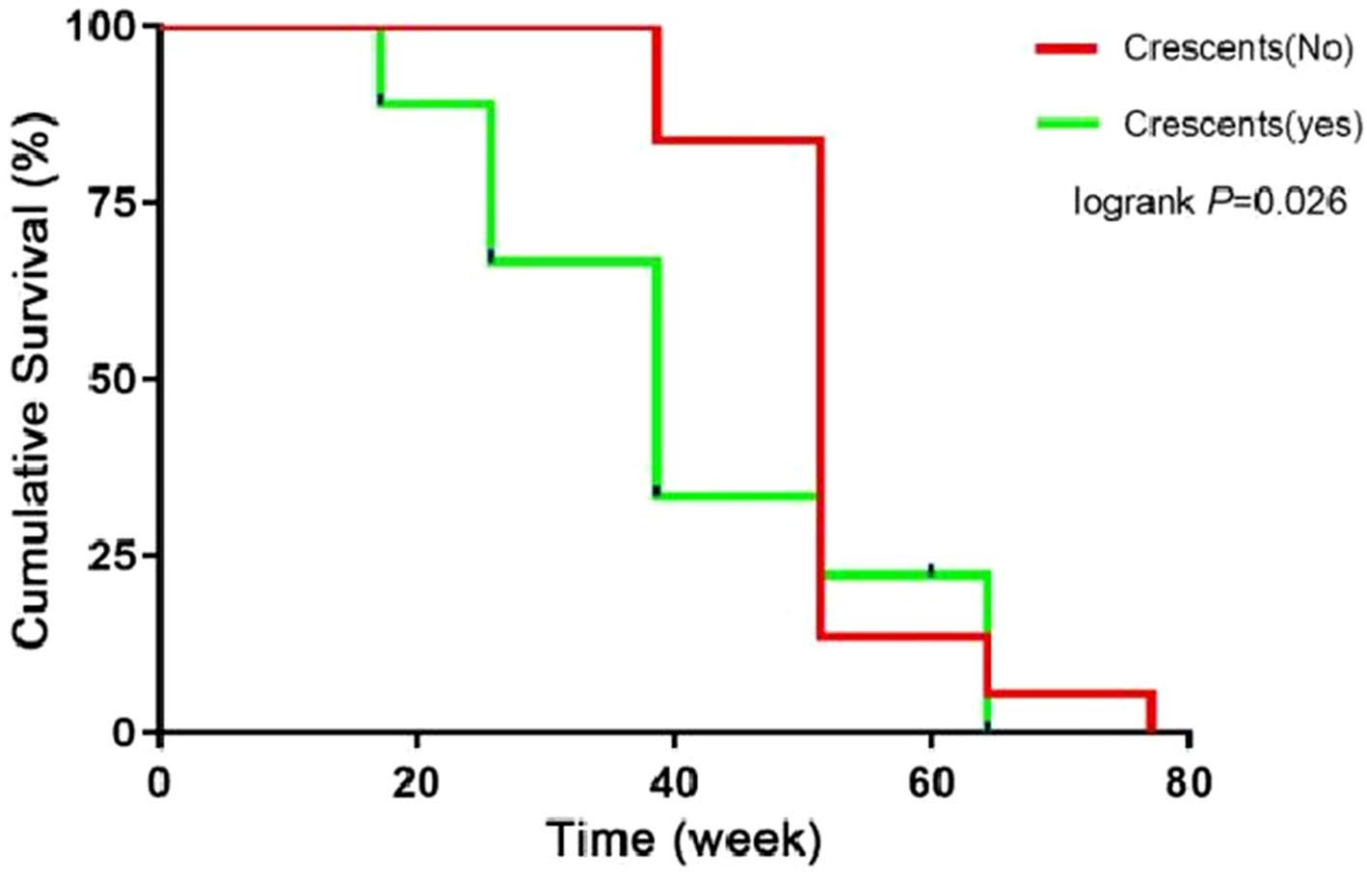
Comparison of the cumulative survival rates of the two groups

Kaplan–Meier survival analysis showed that the presence of a small number of crescents in PMN had a significant effect on the short-term prognosis of patients (Log⁃rank: *P* = 0.026), see Figs. 2 and 3. Crescents (per 1% increase) were an influential factor in patient prognosis (*P* < 0.05), and as this was the only indicator that was significant, a multifactorial survival analysis was not performed. See Table 4, Fig. 2.

**Table 4:** Regression analysis of univariate COX risk proportions affecting patient prognosis

	Single-factor analysis				Multi-factor analysis			
	HR	95% CI		*P*	HR	95% CI		*P*
Group (PMN combined crescent)	0.641	0.284	1.449	0.286				
Gender (female)	1.393	0.702	2.764	0.343				
Age (increased by 1 year)	0.984	0.962	1.007	0.168				
Percentage of crescents (increased by 1%)	119,701.825	49.073	291,986,880	0.003	110,866	27.902	440,512,972.8	0.006
Interstitial inflammatory cell infiltration (present)	1.072	0.147	7.807	0.946				
Interstitial fibrosis and tubular atrophy (yes)	1.269	0.693	2.32	0.44	1.018	0.533	1.942	0.957
UTP (increased by 1 g/24 h)	1.048	0.896	1.225	0.557				
UTP at last follow-up(increased by 1 g/24 h)	1	0.872	1.146	0.999				
eGFR (decreased by 1 mL/min/1.73 m^2^)	0.999	0.99	1.008	0.832				
Finale GFR (decreased by 1 mL/min/1.73 m^2^)	1	0.989	1.011	0.999				
Serum albumin (increased by 1 g/L)	1.03	0.966	1.099	0.368				
Serum albumin at last follow-up (increased by 1 g/L)	0.993	0.961	1.026	0.678				
Nephrotic syndrome	0.906	0.487	1.687	0.756				

*HR* Hazard ratio, *eGFR* estimated glomerular filtration rate

## Discussion

1. Analysis of the incidence of IMN combined with crescent formation Combined crescentic nephropathy is mostly seen in secondary membranous nephropathy. Pathological findings of primary membranous nephropathy combined with the crescent formation in cases of combined blood PLA2R antibodies and positive renal tissue PLA2R are less common [2, 9] and are also reported as cases abroad [10, 11]. In the present study, we retrospectively analysed patients with primary membranous nephropathy in our department over the past 4 years. After excluding secondary factors, such as lupus and vasculitis, we found that the incidence of crescentic MN was 2.86%, and although the incidence was low, the clinical, pathological and prognostic differences compared to PMN also raised our concern.

2. Analysis of clinical features In our study, we found that patients with PMN with crescentic bodies had significantly higher proteinuria and creatinine levels at presentation compared to the simple PMN group, and 90% presented with nephrotic syndrome. Rodruguez et al. [2] reported that crescentic MN usually presented with massive proteinuria and decreased GFR. Wang Shuwei et al. [12] found that patients with crescentic MN (crescentic proportion 7%) had higher proteinuria and serum creatinine levels than the PMN group. This is consistent with their study. However, there is inconsistency with other studies [3, 13]. Considering that the median percentage of crescent bodies in patients in our study was 5.72%, compared to the high percentage of crescent bodies in the latter two studies, the role of this factor cannot be excluded. This leads to the hypothesis that the proteinuria and creatinine levels in membranous nephropathy with crescent formation are relatively severe. In the study, the group found positive expression of anti-PLA2R antibodies and PLA2R in all patients with crescentic MN, with higher antibody titres and higher antigen fluorescence intensity than the control group, but no significant differential expression. The mechanism of crescent formation in PMN is currently unknown, but it is known that PLA2R antibody titres are significantly associated with the development of proteinuria and spontaneous remission [14]. A case report of an elderly female patient with PMN combined with crescent formation (72%) who presented with nephrotic syndrome, haematuria and rapidly progressive renal dysfunction with high serum antibody titres was treated with plasma exchange and melphalan combined with glucocorticoids, and after clearance of anti-PLA2R antibodies, the patient experienced complete resolution of proteinuria and recovery of renal function [15]. With the exclusion of circulating ANCA and anti-GBM effects, we hypothesize a pathogenic role for PLA2R autoimmunity in a crescent formation, but as primary membranous nephropathy with crescent is relatively rare, there are no uniform studies on the diagnosis and mechanism of the disease, and we will expand our sample size for further analysis.

3. Analysis of pathological features In the cohort, we found inflammatory cell infiltrates in 97.44% of PMN patients, with 100% crescentic MN and a heavy interstitial inflammatory cell infiltrate in the kidney. Arrizabalaga et al. [16] reported that individual cases of crescentic MN in patients lacking anti-GBM antibodies, ANCA, lupus or chronic infection also exhibited a large interstitial inflammatory cell infiltrate. Consider a periglomerular inflammatory response that may be triggered by pro-inflammatory mediators released from activated mural epithelial cells within the Bowman's capsule. When there is a significant periglomerular T-cell infiltration, Bowman's capsule encapsulates T cells to prevent the glomerular inflammatory response but promotes fibrous organisation of the crescent and irreversible damage [17]. We thus propose the hypothesis that a cell-mediated inflammatory response may be involved in the transformation of crescentic MN. Tubular atrophy and interstitial fibrosis were also found to be more prominent in the crescentic MN than in the control group, which is consistent with previous studies [3, 18]. Considering the small sample size, further studies with an expanded sample size are still needed in follow-up. In our study, we noted late pathological staging of crescentic MN, but previous studies did not find differential term expression in PMN staging between the two groups, considering the presence of two factors: firstly, the time from onset to renal biopsy expressed by patients in PMN was comparable, but the true specific time of onset was unclear; secondly, there is stability, progression and spontaneous remission in the progression of membranous nephropathy and staging cannot be used as pathogenesis of crescent formation mechanism. In terms of glomerular immunofluorescence, there were differences in C1q and C3 expression in renal tissue between the two groups, and the current study suggests that the classical pathway of complement is activated in some PMN patients and plays an important role in renal pathological injury, possibly leading to more pronounced C3c deposition and more advanced pathological staging of PMN [19]. Some PMN patients with classical pathway activation and C1q (15.38%) expression in renal tissue were also found in our contemporaneous PMN complement-related studies. In the present study, C3 glomerular expression was found to be more pronounced in PMN with crescent bodies, considering the involvement of C3 in crescent body formation in IgA nephropathy and the presence of complement response activation in the latter [20]; whereas previous studies found that there may be local dysregulation of glomerular complement in the formation of crescent bodies in small-vessel vasculitis [21, 22], we consider that complement response may contribute to crescent body formation in PMN, However, the exact mechanism needs to be further investigated.

4. Prognostic analysis—Crescent formation as a factor affecting prognosis In our study we followed the same treatment regimen for both groups according to the 2012 KDIGO Glomerulonephritis Guidelines. At follow-up, we found a poor response to treatment with crescentic MN and a lower remission rate than in the PMN group. Performing KM survival analysis and multifactorial cox analysis, we found that crescent formation and a high proportion of crescentic bodies were risk factors for poor renal prognosis in PMN patients. This is consistent with previous studies [16]. We considered: 1. As both crescentic MN serum and renal tissue are positive for PLA2R expression, patients with crescentogenic MN may be a distinct subgroup of idiopathic MN with autoimmune mechanisms associated with PLA2R. In autoimmune mechanisms, clinical phenotypes have often been reported in association with different epitope responses. Therefore, the autoimmune character of anti-PLA2R antibodies in patients with crescent-forming MN may explain the ineffective treatment.2. With reference to previous studies, the treatment regimen for patients with crescent-forming MN is the same as that for PMN, but it has been shown [3] that patients with PMN accompanied by crescent bodies achieve a lower rate of remission when treated with the same immunosuppressive therapy as patients with PMN without crescent bodies. This, combined with our study demonstrating that crescent formation is an influential factor in the poorer prognosis of patients, is consistent with previous studies in which crescents were patients treated for persistent non-remission. However, there is a paucity of published literature on patients with crescentic MN [2, 23], and there are no treatment recommendations or guidelines for this entity. However, treatment is still recommended to be intensive based on previous rare case reports [15, 19–26]. A patient with PMN combined with crescentic bodies (32 glomeruli, six cellular, five cellular fibrous, one fibrous, and one small cellular crescent formation seen) was identified in our study but was not included in the study cohort considering the coexistence of positive serum anti-GBM antibodies. This patient presented clinically with nephrotic syndrome concomitant with acute kidney injury, no extra-renal manifestations of GBM, serum PLA2R antibody titre 600, PLA2R (3 +) visible on renal biopsy, no nephropathological manifestations of GBM, pathological diagnosis: membranous nephropathy with partial crescent formation. For this patient's clinicopathological and specific antibody manifestations, a low dose hormone shock was administered, followed by adjustment of adequate hormone + The patient achieved partial remission after 3 months, while the serum PLA2R antibody gradually decreased to normal. Considering the sample size limitations, further studies are still needed to determine the advantages and risks associated with this treatment strategy in these patients.

## Conclusion

In conclusion, we report a group of patients with crescentic MN with a mass incidence of approximately 2.86% in the PMN group during the same period, excluding the common etiological conditions of crescentic nephritis. Combined with positive serum and renal tissue PLA2R expression in these patients, it was considered that crescent formation may be PLA2R autoimmune mediated, involving both cellular inflammation and a possible complement response. Also in the study, we found that in these patients, compared to the control group, the patients had high 24 h urine protein quantification, high creatinine on renal biopsy and low eGFR; pathological manifestations were seen in the form of heavy interstitial inflammatory cell infiltration and a high rate of tubular atrophy and interstitial fibrosis; the histopathological staging was later than that of the control group. The same treatment regimen as for PMN was not effective, and crescent formation may be a factor in the lack of remission and the rapid progression of renal function, which may require more aggressive treatment. Also, considering the possibility of crescentic MN as a specific pathological type of PMN, a further subgroup could be set up for a controlled study with PMN at moderate to high risk of progression, however, there are limitations due to the small sample size of this study, which may be subsequently validated by a multicentre study with a large sample.

## Data Availability

All data used for this article are available.
